# Factors influencing the course of post-COVID-19-related symptoms: A bidirectional cohort study among employees in health and welfare services in Germany

**DOI:** 10.3205/dgkh000516

**Published:** 2024-11-05

**Authors:** Lara Steinke, Claudia Peters, Albert Nienhaus, Matthias Bethge, Peter Koch

**Affiliations:** 1 University of Lübeck, Lübeck, Germany; 2 Competence Center for Epidemiology and Health Services Research for Healthcare Professionals (CVcare), Institute for Health Services Research in Dermatology and Nursing (IVDP), University Medical Center Hamburg-Eppendorf (UKE), Hamburg, Germany; 3 Department for Occupational Medicine, Hazardous Substances and Health Sciences (AGG), Institution for Statutory Accident Insurance in the Health and Welfare Services (BGW), Hamburg, Germany; 4 Institute for Social Medicine and Epidemiology, University of Lübeck, Lübeck, Germany

**Keywords:** COVID-19, post-acute COVID-19 syndrome, post-COVID-19 syndrome, health personnel, social workers, persistent symptoms, follow-up, time to symptom-free, risk factors

## Abstract

**Objective:**

The aim of this study was to determine the prevalence and trajectory of persistent symptoms following COVID-19 and to investigate factors influencing these among employees in the health and welfare services in Germany.

**Methods:**

This exploratory, mixed retro- and prospective cohort study using paper-and-pencil questionnaires was conducted among insured persons of the German Social Accident Insurance Institution for the health and welfare services with a SARS-CoV-2 infection in 2020. The baseline survey in February 2021 was succeeded by two follow-up surveys after 8 and 13 months. Demographic data, information on the acute illness and persistent symptoms were collected. Kaplan-Meier curves were created to visualize the course of recovery. Factors influencing the time to recovery were analyzed using multivariate Cox regressions.

**Results:**

Of the 4,325 people contacted, 2,053 took part in the survey (response rate: 47%). 1,810 people were included in the analysis. The most common persistent symptoms at all three survey time points were fatigue, concentration and memory problems, and dyspnea. After three months, 76.2% (95% CI: 74.2–78.2%) of participants still reported symptoms, after 18 months this dropped to 67.2% (95% CI: 65.0–69.4%). Significant risk factors for persistent symptoms were female sex (HR: 0.72; 95% CI: 0.58–0.88), age over 50 years (HR: 0.63; 95% CI: 0.50–0.78), a higher number of pre-existing illnesses and a higher number of severe acute symptoms. Respiratory and hormone-metabolic pre-existing conditions as well as severe dyspnea, smell or taste disorders, fatigue and memory or concentration problems during the acute COVID-19 illness also reduced the probability of complete recovery. Compared to other professions, working as a doctor had a protective effect (HR: 1.42; 95% CI: 1.11–1.80).

**Conclusion:**

More than a year after a COVID-19 illness, two-thirds of the healthcare staff surveyed reported persistent symptoms. This high number emphasizes the importance of long-term consequences of the COVID-19 pandemic for public health and the need for suitable therapy and rehabilitation concepts, especially for healthcare staff with post-COVID syndrome.

## Zusammenfassung

**Zielsetzung:**

Ziel der Arbeit war die Ermittlung der Prävalenz und Verläufe von anhaltenden Symptomen nach einer COVID-19-Erkrankung sowie die Untersuchung von Einflussfaktoren auf diese unter Beschäftigten im Gesundheitsdienst und der Wohlfahrtspflege in Deutschland.

**Methode:**

Die explorative, gemischt retro- und prospektive Kohortenstudie mittels Paper-Pencil-Befragung erfolgte unter Versicherten der Berufsgenossenschaft für Gesundheitsdienst und Wohlfahrtspflege (BGW) mit einer SARS-CoV-2-Infektion im Jahr 2020. Auf die Befragung im Februar 2021 folgten zwei Follow-up-Befragungen nach acht bzw. 13 Monaten. Erfragt wurden demographische Daten, Informationen zur Akuterkrankung sowie zu anhaltenden Symptomen. Zur Darstellung des Genesungsverlaufs wurden Kaplan-Meier-Kurven erstellt. Einflussfaktoren auf das Persistieren von Symptomen wurden mit multivariaten Cox-Regressionen geprüft.

**Ergebnisse:**

Von 4.325 angeschriebenen Personen nahmen 2.053 an der Befragung teil (Response-Rate: 47%). 1.810 Personen wurden in die Analysen eingeschlossen. Die häufigsten persistierenden Symptome zu allen drei Befragungszeitpunkten waren Müdigkeit und Erschöpfung, Konzentrations- und Gedächtnisprobleme sowie Kurzatmigkeit. Nach zwölf Wochen berichteten noch 76% (95% Konfidenzintervall (95% CI): 74–78%) von Symptomen, nach 18 Monaten 67% (95% CI: 65–69%). Signifikante Risikofaktoren für persistierende Symptome waren weibliches Geschlecht (Hazard Ratio (HR): 0,72; 95% CI: 0,58–0,88), Alter über 50 Jahre (HR: 0,63; 95% CI: 0,50–0,78), höhere Anzahl an Vorerkrankungen und schweren Akutsymptomen. Auch respiratorische und hormon-metabolische Vorerkrankungen sowie schwere Kurzatmigkeit, Geruchs- oder Geschmacksstörungen, Fatigue und Gedächtnis- oder Konzentrationsprobleme während der akuten COVID-19-Erkrankung reduzierten die Genesungswahrscheinlichkeit. Im Vergleich zu anderen Berufen hatte ärztliche Tätigkeit einen protektiven Effekt (HR: 1,4; 95% CI: 1,11–1,80).

**Fazit:**

Über ein Jahr nach einer COVID-19-Erkrankung berichtete der Großteil des befragten Gesundheitspersonals von anhaltenden Symptomen. Dieser hohe Anteil unterstreicht die Bedeutung der Langzeitfolgen der COVID-Pandemie für die öffentliche Gesundheit sowie die Notwendigkeit geeigneter Therapie- und Rehabilitationskonzepte, insbesondere für betroffenes Gesundheitspersonal.

## Background

Coronavirus Disease 2019 (COVID-19) mainly affects the respiratory tract but can also lead to symptoms in various organ systems. Aside from asymptomatic infections and mild illnesses, severe and fatal courses of the disease can occur. While many occupational groups avoided infection by working from home and isolating themselves in everyday life, healthcare workers were exposed to a particularly high risk of infection when caring for patients, especially those suffering from COVID-19. Compared to other professions, healthcare workers were therefore more often infected with Severe Acute Respiratory Syndrome Coronavirus Type 2 (SARS-CoV-2) and were particularly frequently affected by work-related infections [1], [2].

However, as has been observed with other viruses [3], [4], a SARS-CoV-2 infection can not only lead to an acute illness, but can also result in persistent symptoms that last for weeks to years. First reports of persistent health restrictions following a SARS-CoV-2 infection were made early on in the pandemic by those affected, who described themselves as “long haulers” [5]. Other terms used in the literature are long COVID, post-acute sequelae of SARS-CoV-2 infection (PASC) or post-COVID syndrome. The World Health Organization (WHO) defines a post-COVID-19 condition as a condition that usually occurs three months after a confirmed or probable SARS-CoV-2 infection with symptoms persistent for at least two months, which cannot be explained otherwise, and which generally affect daily activities. Those symptoms can reappear after an initial recovery or outlast the acute COVID-19 disease and persist permanently or have a fluctuating course [6]. The British National Institute for Health and Care Excellence (NICE) defines post-COVID-19 syndrome as symptoms lasting longer than twelve weeks. Symptoms lasting four to twelve weeks are labelled by NICE as ongoing symptomatic COVID-19 [7]. The symptoms of post-COVID include fatigue, dyspnea, taste or smell disorders and cognitive impairment [8]. They can lead to severe impairments in quality of life and restrictions in the ability to work, including occupational disability [8]. Possible causes discussed include endothelial dysfunction, viral persistence, autoimmunity, persistent inflammation, Epstein-Barr virus reactivation and psychosocial factors [9], [10]. Although people with severe acute illness have a higher risk of post-COVID, people with mild acute illness can also be affected by post-COVID.

Due to the heterogeneous study situation with large differences in design, observation time, study population and post-COVID definition, it is still difficult to estimate the prevalence of post-COVID-19. Systematic reviews, which included studies among both hospitalized and non-hospitalized patients, estimate the prevalence of post-COVID after twelve weeks at 52% to 59% [11], [12]. If only non-hospitalized patients were examined, the proportion was between 6% and 51% [13], [14], [15]. Two controlled, population-based, prospective cohort studies, one of which also considered the symptom burden prior to SARS-CoV-2 infection, estimated the prevalence of post-COVID at 11% [16], [17]. These figures mostly refer to people who were unvaccinated at the time of infection. With millions of people infected with SARS-CoV-2 throughout Germany [18], these long-term consequences not only lead to a major burden for the affected individuals, but also pose a considerable problem for the public health system and the economy.

Healthcare staff were particularly frequently affected by post-COVID [19]. There are, however, only a few longitudinal and mostly small studies among healthcare personnel. Due to the prominent role of healthcare workers in coping with COVID-19, but also their particular risk of being affected by post-COVID, the aim of this study is to determine the prevalence and symptom trajectories of post-COVID-19 and the factors influencing them over a period of more than one year among employees in the health and welfare services in Germany.

## Methods

### Study design and setting

This exploratory, bidirectional cohort study using paper-and-pencil questionnaires was conducted among insured persons of the German Institution for Statutory Accident Insurance in the Health and Welfare Services (BGW). The BGW is a statutory accident insurance for employees of non-governmental healthcare and welfare organizations. The baseline survey (T1) took place in February 2021. A descriptive analysis of the baseline survey without non-responder analysis is given by Peters et al. [20]. Non-responders received a one-off reminder letter in April 2021. A short non-responder questionnaire asked about the course of symptoms and the reason for the refusal to participate. Two follow-up surveys (T2, T3) were conducted among the responders 8 and 13 months after the initial survey. Manuscript drafting followed the recommendations of the STROBE statement [21]. The study was approved by the ethics committee of the Hamburg Medical Association (2021-10463-BO-ff). All participants gave their informed written consent to take part in the study.

### Participants

All insured persons from two districts in Germany (Cologne and Dresden) with a suspected occupational SARS-CoV-2 infection by December 31, 2020 were contacted. A positive PCR or antigen test was required for inclusion. Non-symptomatic infections were not subject to mandatory reporting. However, symptoms during the infection were not part of the inclusion or exclusion criteria of the study. Exclusion criteria for participation were the absence of a SARS-CoV-2 infection, limited reading and writing skills and limited German-language skills. Respondents whose SARS-CoV-2 infection was asymptomatic were excluded from the present analysis. We also excluded participants who did not provide information on the date of the positive SARS-CoV-2 test or the time to recovery asked in the baseline survey. 

### Variables and data sources

The baseline-questionnaire contained items on socio-demographic data, height, weight, smoking status, physical exercise habits, subjective state of health and occupational information. Pre-existing medical conditions and retrospective data on the acute COVID-19 disease were recorded. For better presentation and to test the assumption of proportional hazards for Cox regression, the variables “age”, “body mass index (BMI)”, “number of pre-existing conditions”, and “number of severe acute symptoms” were categorized (see Table 1 (Tab. 1) and Table 2 (Tab. 2)). The variable “occupational task” was categorized into the groups “medical activity” and “non-medical activity”. A total of 13 acute symptoms were presented as a list. Respondents could rate their symptoms as “not present”, “mild”, “moderate” and “severe”. Due to the low correlation between the items, a planned dimensionality reduction of the acute symptoms using principal component analysis was abandoned. Instead, the number of severe acute symptoms was added up to a sum score. 

Questionnaires 2 and 3 asked for information on vaccination and reinfections.

The presence of persistent symptoms was recorded at all three survey times. If persistent symptoms were present, these were queried in the same way as acute symptoms. The symptoms “sleep disorders”, “lack of drive”, “hair loss” and “dizziness” were only recorded in the second and third survey, the symptom “limited exercise capacity” only in the third survey. The symptom “limb pain” of the first questionnaire was modified into “muscle and limb pain” in the two subsequent questionnaires. All other symptoms were included in all three questionnaires. If no persistent symptoms were present, the time to recovery could be specified in days or weeks in questionnaire 1. In questionnaires on T2 and T3, respondents could choose between the categories “up to 4 weeks”, “up to 3 months”, “up to 6 months”, “up to 12 months” and “longer than 12 months”. 

### Statistical methods

The presentation of persistent symptoms at the different survey times was conducted among respondents who took part in all three surveys. This analysis did not include people who reported symptoms again in a later questionnaire after reporting recovery. The differences between the survey time points were tested for significance using the McNemar test for paired samples. This was done between T1 and T3, or between T2 and T3 if the symptom was not asked about at T1. 

A survival analysis was performed among all included participants. The recovery from all symptoms was defined as the event. People who became symptomatic again after reporting recovery were only included in the survival analysis up to the first event. The observation time was defined as the time from the positive SARS-CoV-2 test to recovery or censoring. For T1, this corresponds to the times given by the participants. At T2 and T3, the time to recovery was defined as the mean time in the specified interval. This resulted in an observation period of 14 days for the “up to 4 weeks” category. For the “up to 3 months” category, it was 60 days, for the “up to 6 months” category 135 days and for the “up to 12 months” category 270 days. For the category “longer than 12 months”, the observation period was calculated as the average time between 365 days and the date of receipt of the last questionnaire completed. The survival functions were calculated using the Kaplan-Meier method and visualized using Kaplan-Meier curves. Group differences were tested using log-rank tests. In addition, multivariate Cox regressions were estimated. The proportional risk assumption was assessed using log-minus-log plots. If the course of the log-minus-log plot was unclear, a time-dependent interaction term was added in the Cox regression model. If this interaction term was non-significant, the proportional risk assumption for the variable was assumed. The variables considered in the multivariate cox regressions were: “sex”, “age”, “BMI”, “pre-existing conditions”, “smoking”, “children”, “living situation”, “employment status”, “occupational task”, “workplace”, and “severe acute symptoms”. Several Cox regression models were designed to test both the influence of the individual pre-existing conditions and their number, as well as the influence of the severe acute symptoms and their number. As less than 50% of respondents had an event during the observation period, the median time to freedom of symptoms could not be reported. Instead, the time to symptom-freedom of 25% of the respondents is reported. 

It was not possible to examine the influence of vaccinations on the time to recovery due to a lack of temporal data and therefore an inaccurate allocation of vaccination dates. Among the participants with a SARS-CoV-2 reinfection, no event was reported within the observation period. The variable “reinfection” could therefore not be included in the Cox regression. Instead, a group comparison using the chi-squared test was performed. A drop-out analysis was performed using multiple logistic regression. The variables were selected using a stepwise backward selection.

Sample characteristics are presented as mean with standard deviation (SD) or median with interquartile range (IQR) for continuous variables and as numbers and percentages for categorical variables. The data were analyzed using SPSS version 27. The significance tests were two-sided at a significance level of 0.05.

## Results

### Participation and reasons for non-participation

**Figure 1:**
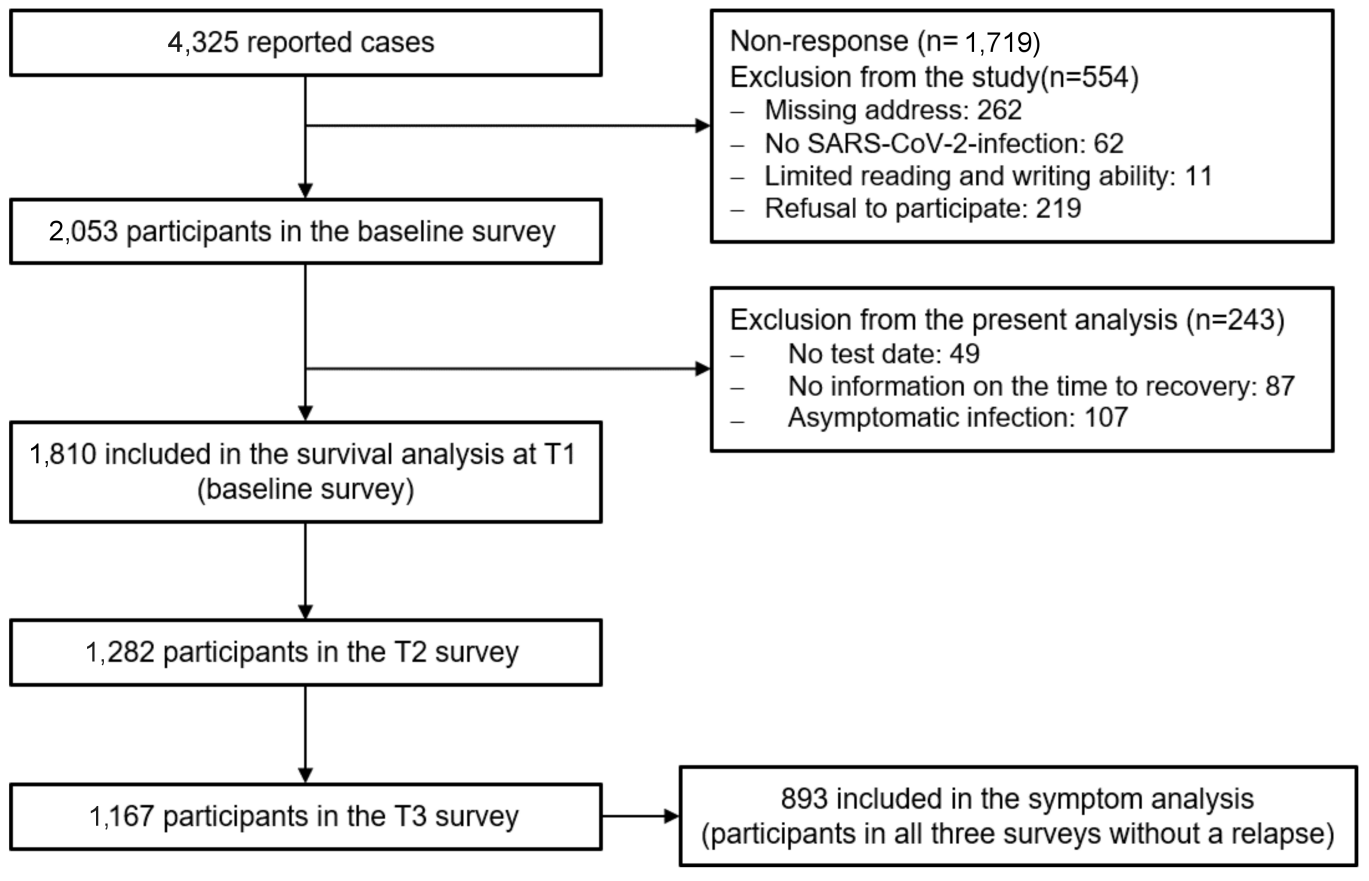
Flowchart of the study program

Of the 4,325 insured persons contacted, 2,053 took part in the survey (response rate=47.5%). Of these, 554 were excluded from the study and 243 were excluded from the present analysis (see Figure 1 (Fig. 1)). Therefore, a total of 1,810 respondents were included in the analyses. Of these, 1,282 participants took part in the first and 1,167 in the second follow-up survey (follow-up rate: 70.8% and 64.5% respectively). A total of 997 participants took part in all three surveys. The reasons for non-response given in the non-responder questionnaire with 310 participants included a lack of time (18.1%) or interest (12.3%), absence of symptoms (12.6%), no previous SARS-CoV-2 infection (12.6%), personal reasons (7.7%) and other reasons (32.0%). In the drop-out analysis young age, smoking and poor health in the baseline survey were identified as significant risk factors for drop-out. The R2 according to Nagelkerke of the final model was 7%. The median time from the positive SARS-CoV-2 test to receipt of the questionnaire was 301 days (IQR: 154–368) for the baseline survey (T1), 516 days (IQR: 365–589) for the first follow-up survey (T2) and 669 days (IQR: 516–730) for the second follow-up survey (T3).

### Participants’ characteristics

Of the included participants, 1,489 (82.3%) were female. The mean age was 48.0 (SD: 12.2) and the mean BMI was 27.0 (SD: 5.8). 275 (15.4%) participants reported smoking and 531 (29.9%) reported an absence of regular physical activity. Most respondents (1,127; 62.5%) worked in nursing. Pre-existing conditions were stated by 64.0% (1,159). On average, the number of pre-existing conditions quoted by participants with at least one pre-existing conditions was 1.8 (SD: 1.0). For detailed participant characteristics see Table 1 (Tab. 1).

### Acute disease

The presence of a SARS-CoV-2 infection was confirmed in 90.7% (1642) of cases by a positive PCR test, in 1.4% (26) by a positive antigen test and in 7.3% (132) by both test methods. Most positive tests were dated to spring or autumn 2020. On average, 2.7 (SD: 2.4) of the 13 acute symptoms surveyed were reported as severe. The symptoms most frequently reported in severe form were “fatigue”, “smell or taste disorder”, and “limb pain” (Table 2 (Tab. 2)). Medication for the treatment of acute COVID-19 disease was taken by 1,045 (58.1%) participants. Most of these were non-steroidal anti-inflammatory drugs. 33.5% (606) of respondents received outpatient medical care due to acute COVID-19 disease. 7.3% (132) were hospitalized, 35 (1.9%) received intensive medical care and 13 (0.7%) were ventilated (Table 2 (Tab. 2)).

### Vaccinations and relapses

Of the participants of the last survey (T3; n=1,167), 1,082 (92.7%) had been vaccinated at least once and 836 (71.6%) three or four times. 10 (0.9%) respondents were unvaccinated and 75 (6.4%) did not provide any information. It can be assumed that all participants were unvaccinated at the time of first infection, as the date of first infection of almost all participants was before the authorization of the first vaccine in Germany. Among the participants of the third survey (T3; n=1,167), 133 (11.4%) people reported a relapse, i.e., recurrence of symptoms after a symptom-free interval.

### Persisting symptoms

**Figure 2:**
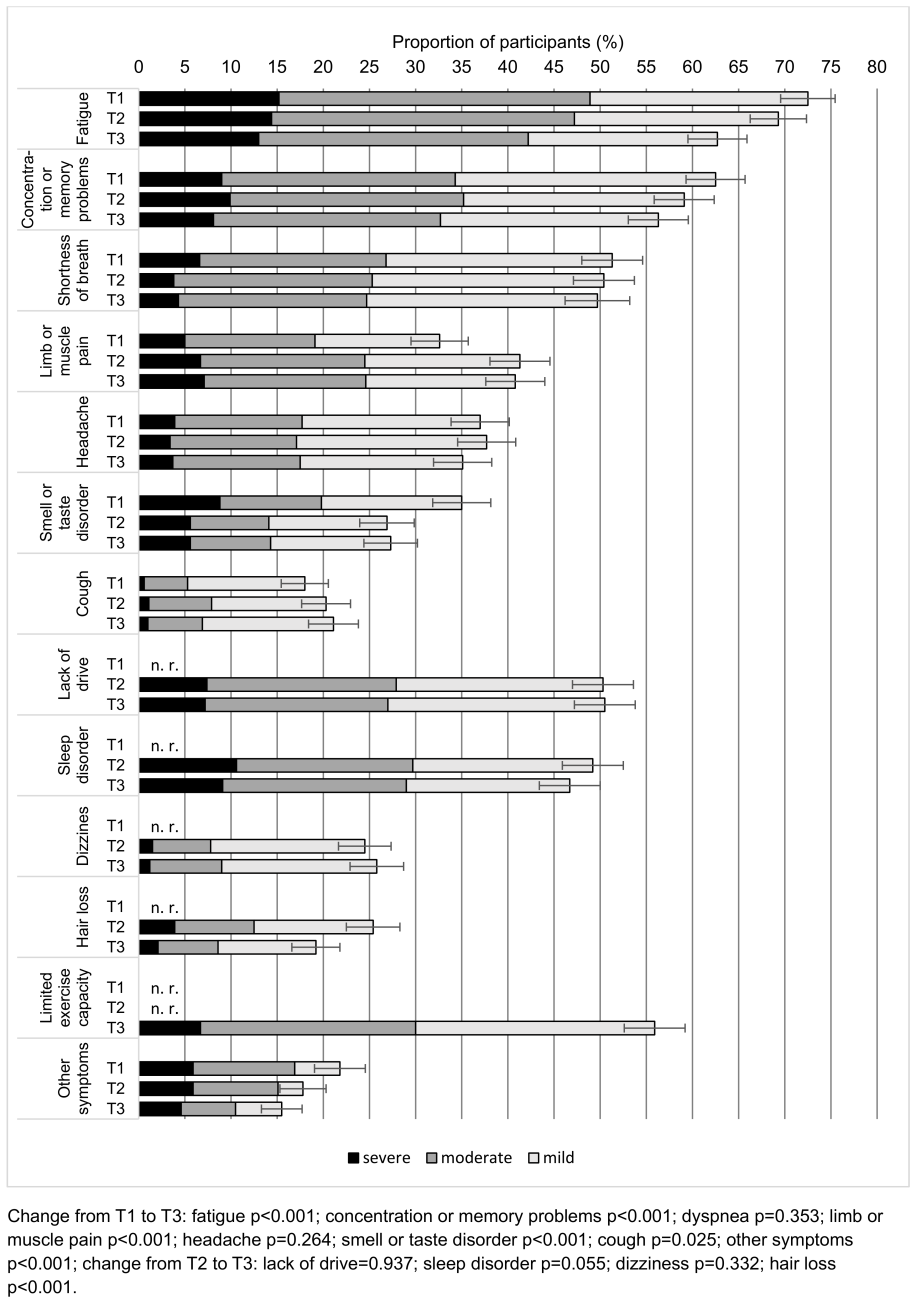
Course of persistent symptoms among participants of all surveys and without relapses (n=893) nested by symptom severity. The error bars indicate the 95% CI. n. r.: symptom not recorded at this survey time point

Among the 893 respondents who took part in all three surveys and did not have a relapse 764 (85.7%, 95% confidence interval [CI]: 83.4–88.0%) reported persistent symptoms at time point T1. At time points T2 and T3, the figures were 694 (77.7%, 95% CI: 75.0–80.4%) and 635 (71.1%, 95% CI: 68.1–74.1%) respectively. The progression of the individual symptoms in the subgroup described is shown in Figure 2 (Fig. 2). At all three survey times, the most common symptom of any severity was “fatigue”, followed by “concentration or memory problems”. “dyspnea”, “lack of drive”, “sleep disorders” and “limited exercise capacity” were also frequently mentioned. The proportion of those with “fatigue”, “concentration or memory problems” and “smell or taste disorders” decreased significantly between T1 and T3 (all p<0.001). For “hair loss”, which was not asked at T1, a significant decrease was observed between T2 and T3 (p<0.001). A significant increase from T1 to T3 was shown for the symptoms “cough” (p=0.025) and “muscle and limb pain” (p<0.001). No statistically significant change could be demonstrated for the remaining symptoms.

### Survival analysis

**Figure 3:**
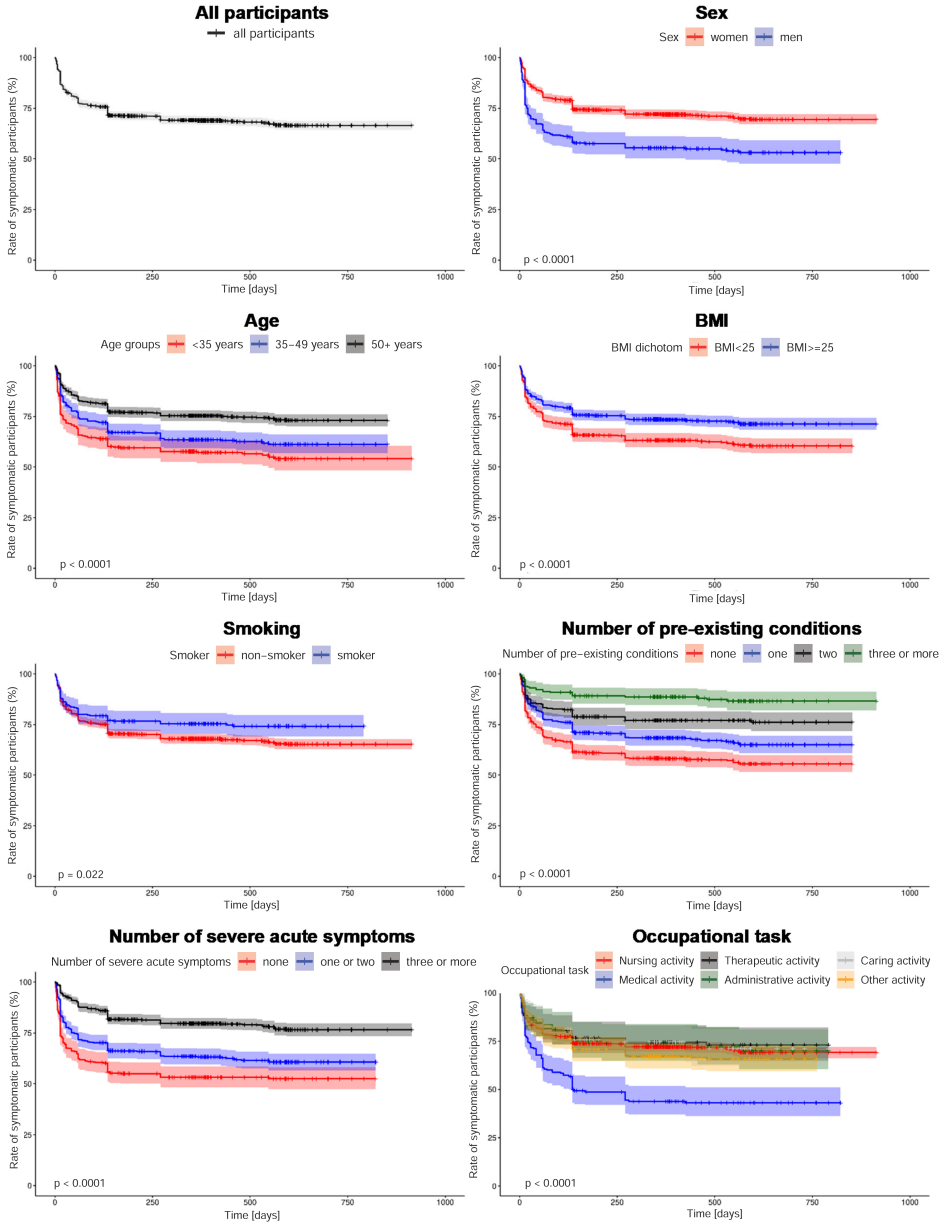
Kaplan-Meier curves p-values: log-rank-test

The Kaplan-Meier curves are shown in Figure 3 (Fig. 3). Among all participants, 82.8% (95% CI: 81.0–84.6%) were still symptomatic after four weeks and 76.4% (95% CI; 74.4–78.4%) after twelve weeks. After five months, the proportion of symptomatic participants had fallen to 71.4% (95% CI: 69.2–73.6%). Thereafter, this proportion decreased only very slowly. After twelve months, 69.0% (95% CI: 66.8–71.2%) of respondents were still affected by persistent symptoms. After 18 months this decreased to 67.2% (95% CI: 65.0–69.4%). The time to recovery for 25% of the participants was four and a half months. Among the participants who became symptom-free, the median time from positive SARS-CoV-2 test to recovery was 28 days (IQR: 12–112). 

When testing the proportional risk assumption, overlaps or inconclusive results were found in the log-minus-log plots of the variables “smoking”, “number of pre-existing conditions”, “obesity”, “skin diseases” and “hormonal diseases”. When including these variables as time-dependent interaction terms in the Cox regression, no significant results were found. Therefore, the proportional risk assumption for the variables mentioned was presumed. For all other variables, the proportional risk assumption could be made based on the log-minus-log plots.

Several Cox regression models were created (Table 3 (Tab. 3)). While model 1 contains the number of pre-existing conditions and the number of severe acute symptoms, model 2 includes the detailed severe acute symptoms and model 3 the detailed pre-existing conditions. The final model contains both the detailed pre-existing conditions and the detailed severe symptoms during the acute COVID-19 illness. As the models were statistically equivalent, we decided in favor of the final model due to the most detailed presentation.

In the final model, female sex (Hazard Ratio [HR]: 0.72; 95% Cl: 0.58–0.88), age >50 years (HR: 0.63; 95% CI: 0.50–0.78) and the presence of pre-existing “respiratory diseases” (HR: 0.63; 95% CI: 0.45–0.87) and “hormonal or metabolic diseases” (HR: 0.72; 95% CI: 0.57–0.91) were identified as significant risk factors for symptom persistence. Among the severe acute symptoms, a reduced probability of recovery was observed for “dyspnea” (HR: 0.68; 95% CI: 0.50–0.93), “smell or taste disorder” (HR: 0.83; 95% CI: 0.70–0.99), “fatigue” (HR: 0.62; 95% CI: 0.50–0.77) and “memory or concentration problems” (HR: 0.60; 95% CI: 0.44–0.82). Compared to other occupations, an increased probability of recovery was shown for “medical activity” (HR: 1.42; 95% CI: 1.11–1.80). No significant influence was found for “obesity”, “smoking”, “children”, “living situation”, “employment status”, “physical exercise” and “workplace”.

In the models 1 to 3, the probability of recovery decreased significantly with an increasing number of pre-existing conditions or severe acute symptoms. In model 1, the chance of recovery decreased by 19% (HR: 0.81; 95% CI: 0.67–0.98) if one pre-existing condition was stated instead of none. For two pre-existing conditions, this was 39% (HR: 0.61; 95% CI: 0.47–0.80), for more than two 65% (HR: 0.35; 95% CI: 0.23–0.52). If one or two severe symptoms were present during the acute illness, the probability of recovery was reduced by 27% (HR: 0.73; 95% CI: 0.60–0.90). With more than three acute symptoms, this was 59% (HR: 0.41; 95% CI: 0.33–0.51). All other observations remained largely constant in terms of quality and quantity across the models.

### Reinfections

Of the 1,167 participants who took part in the last survey (T3), 245 (21.0%) reported at least one reinfection with SARS-CoV-2. In 92 participants, this reinfection occurred after recovery and therefore could not have had any influence on the time to recovery. Among the 153 remaining respondents with a reinfection, no one reported a full recovery by the last survey. Among the participants without a reinfection or with a reinfection after recovery, 41.6% did fully recover. Compared to participants without a reinfection, the recovery rate among participants with a reinfection was significantly lower (p<0.001). 

### Main findings

In the bidirectional cohort study of more than 1,800 healthcare workers with a SARS-CoV-2 infection in 2020, we were able to show that even 18 months after infection, two-thirds were still affected by at least one persistent symptom. Common persistent symptoms were fatigue, cognitive symptoms, and dyspnea. As risk factors for symptom persistence we identified female sex, older age, a high number of pre-existing conditions, pre-existing respiratory or hormonal-metabolic illnesses, a high number of severe acute symptoms and the presence of severe dyspnea, smell or taste disorders, fatigue, or concentration problems during the acute COVID-19 disease.

## Discussion

### Symptom prevalence

The symptom prevalence for twelve weeks after a SARS-CoV-2 infection (76.4%; 95% CI 74.4–78.4%), fulfilling the NICE criteria for post-COVID among the healthcare workers surveyed, was higher than the prevalence of post-COVID among the general population reported in the literature, which ranges between 10% and 50% [13], [14], [16], [17]. The rate of symptomatic participants after twelve months was 69.0% (95% CI 66.8-71.2%) and therefore also exceeded the values of 54% and 57% reported in two systematic reviews [11], [22].

Possible reasons for the high prevalence in our cohort include both methodological and content-related influences. For several methodological reasons, the prevalence may have been overestimated in our study. First, we excluded asymptomatic patients during infection for whom a lower risk of post-COVID could be shown [23]. Second, contrary to the WHO definition of post-COVID-19 condition, we did not exclude other causes of the symptoms. In a study by Ballering et al. [16], 41% of respondents reported persistent symptoms three to five months after SARS-CoV-2 infection. After accounting for pre-existing symptoms and symptom prevalence among SARS-CoV-2-negative controls, the prevalence of SARS-CoV-2-related persistent symptoms was only 13%. In other studies, examining healthcare workers, the proportion of people with persistent symptoms was 15 to 70% [24], [25], [26], [27]. After accounting for SARS-CoV-2 negative controls, the proportion dropped to 3 to 30% [24], [25], [26], [27]. By instructing participants in the questionnaire to only report symptoms that are a consequence of COVID-19, we aimed to minimize the number of symptoms caused by other diseases. Nevertheless, the blurred distinction between COVID-related and non-COVID-related symptoms may have led to an overestimation of symptom prevalence in our study. Thirdly, unlike other studies and in contrast to the WHO definition, we considered persistent symptoms of any severity and did not presuppose any limitations in everyday life.

In terms of content, it should be noted that the cohort analyzed in this study was not a population-based sample, but rather focused on workers in the healthcare and welfare sectors. This group is characterized by demographic features with a high proportion of women and middle-aged people, with the female sex being a risk factor for post-COVID in our study as well as others [28], [29], [30]. Furthermore, the group studied is characterized by special working conditions with a high frequency of contact with patients and high psychological stress, especially during the COVID-19 pandemic. Healthcare workers are particularly often affected by post-COVID-19 [19]. One reason for this is the high rate of SARS-CoV-2-infections among healthcare workers [31]. In addition to the direct consequences of an infection, social, psychological, and environmental factors might play a role in the development of post-COVID [32]. Psychological stress before the pandemic was identified as a risk factor for post-COVID-19 in some studies [30], [33]. Another reason for the high prevalence of post-COVID among healthcare workers in our study might therefore be the high level of stress during the pandemic.

### Course of symptoms

We observed a rapid decrease in the prevalence of persistent symptoms in the first five months from 100% to 71.4%, while in the following 13 months it decreased by only 4.2%. Similar trends, with a rapid decline in the first six months and little change in the following six to twelve months, were also observed in other studies [34], [35].

### Specific symptoms

Among the symptoms surveyed, fatigue, concentration or memory problems, lack of drive, sleep disorders, dyspnea as well as reduced exercise capacity were the most common, ranging between 47% for sleep disorders and 63% for fatigue. This is consistent with data from systematic reviews and the UK Office for National Statistics [11], [12], [19]. These symptoms are also common in the general population and may have been exacerbated by the general effects of the pandemic situation. However, in a recently published systematic review, which included 23 controlled studies, an increased risk of fatigue, dyspnea, concentration and memory problems was shown among SARS-CoV-2 infected individuals four or more weeks after their infection [36]. 

Except for the symptoms lack of drive and sleep disorders, we did not observe the stagnation or even increase in neurocognitive symptoms described in many other studies [10], [11], [37]. However, the decrease in fatigue and concentration or memory problems did not exceed 10 percent points. Correspondingly the rate of those symptoms remained at a high level with more than 60% of respondents reporting fatigue and more than half reporting concentration or memory problems in the last survey. The stagnation of dyspnea and headaches we observed was already shown by other studies [11], [38], [39], [40]. The increase in the symptoms of muscle and limb pain and coughing is not consistent with the literature. In the case of muscle and limb pain, methodological reasons for the increase are likely, as the symptom was only queried as “limb pain” in the baseline questionnaire, but as “muscle and limb pain” in the following two questionnaires.

### Influencing factors unrelated to acute COVID-19 disease

The association between female sex, older age and higher risk of persistent symptoms is consistent with the literature, even though the influence of older age was not shown in some studies [28], [29], [30]. Some studies also describe an increased risk for middle age [9], [30]. As only 2.2% of the respondents in our cohort were over 65 years old, the influence of older age can only be assessed to a limited extent. No significant influence for smoking and overweight on symptom persistence could be shown in our study.

We further identified a high number of pre-existing conditions as risk factors for persistent symptoms. Particularly respiratory and hormonal-metabolic comorbidities had an influence. In a recently published systematic review, respiratory diseases such as asthma and COPD or the hormonal-metabolic disease diabetes were also identified as risk factors for post-COVID [28]. The same applies to a large prospective cohort study, which also observed a significant influence of the number of existing comorbidities [23].

Compared to other occupational groups, being a physician had a “protective effect” on the persistence of symptoms. The reason for this is unclear and cannot be conclusively clarified in this study. In a study of healthcare workers, Strahm et al. [24] found an increased risk for nursing staff compared to other occupational groups, but no influence for medical work. The frequency of patient contact had no significant influence in that study [24]. Possible influencing factors to be considered include level of education and socio-economic status, for which some studies have shown a significant influence [41], [42], but also job satisfaction. Even before the pandemic, doctors were less likely to be on sick leave than other occupational groups [43]. The reasons for this are also unclear.

### Influencing factors related to acute COVID-19 disease

The number of severe acute symptoms can serve as a surrogate parameter for the severity of the acute illness. We found an increased risk of persisting symptoms with a higher number of severe acute symptoms. This is consistent with the results of systematic reviews that identified hospitalization due to COVID-19 or a more severe acute illness as a risk factor for post-COVID [11], [28]. Studies that, like ours, analyzed the number of acute symptoms also found an increased risk with a higher number of symptoms [8], [24], [34], [44].

The presence of dyspnea, fatigue, concentration problems and severe smell or taste disturbances in the acute phase were associated with an increased risk of persistent symptoms in our cohort. For dyspnea and fatigue this influence has already been described in other studies [35]. These symptoms were also frequently reported as persistent after the acute disease in our cohort. Although smell or taste disturbances were reported less frequently as a persistent symptom, their proportion was particularly high in various studies among SARS-CoV-2 infected persons compared to controls[23], [45], [46]. These symptoms therefore not only persist for a long time, but are also more frequently present in the acute phase among those affected by post-COVID. Why these symptoms persist in post-COVID-19 is still largely unclear, as is the impact of the course of the acute disease and the pathological mechanism.

### Influence of reinfections

We found a significantly lower recovery rate among people with a SARS-CoV-2 reinfection. It can therefore be assumed that there is an increased risk of symptom persistence due to reinfection. This is consistent with the findings of other studies [10], [47].

### Consequences of persistent symptoms

Persistent symptoms have far-reaching consequences for the individual, but also for public health and the labor force. Post-COVID sufferers are more likely to be restricted in their daily lives and report a poorer health-related quality of life than controls [19], [23], [40]. According to a cross-sectional study by Davis et al. [38], 45% of people with persistent symptoms had to reduce their working hours, while a further 22% stated that they no longer worked at all due to their illness. A reduced health-related quality of life and a reduced subjective ability to work among people with persistent symptoms was also found by Peters et al. [20] in the baseline survey of our cohort. In a further analysis of the baseline survey of our cohort, Haller et al. [48] were able to show that this is particularly true for those affected by severe post-COVID fatigue.

### Strengths and limitations

The strengths of the study are the sample size of more than 1800 people and the long follow-up period of almost two years. By using insurance data, we were able to contact all reported SARS-CoV-2 cases from different institutions in two regions of Germany. The response rate of 47% was good, especially in the context of occupational surveys. It can therefore be expected that the sample is a good representation of the analyzed group. The follow-up rates of 71% and 65% were also relatively high. Low age, smoking and poor health in the baseline survey were associated with a higher probability of drop-out. However, with a R^2^ according to Nagelkerke of 7%, only a small proportion of the drop-outs can be explained by these influencing factors, suggesting that they only have a minor impact on our results.

Nevertheless, we must consider the following limitations. First, people without persistent symptoms were probably less interested in participating (selection bias). Second, the partial retrospective data collection may have led to a recall bias, since subjectively perceived symptoms were surveyed and no objectifiable findings were collected. Third, the data from the survival analysis is partially imprecise, as questionnaires 2 and 3 only roughly categorized the time to recovery. Fourth, we did not include a control group in our study. This meant that the prevalence of symptoms in the general population could not be taken into account. Other factors influencing the prevalence are discussed in the relevant section. Fifth, our study is an observational study. The identified influencing factors therefore only show correlations that do not necessarily have a causal relationship. The influence of unrecorded confounding factors cannot be ruled out either. Sixth, as the people studied were infected with SARS-CoV-2 during the period of dominance of the wild type and the alpha variant, our results can only be transferred to other virus variants to a limited extent. The same applies when transferring the results to people who were vaccinated before the initial infection.

## Conclusion

The high prevalence of persistent symptoms we have observed among healthcare and welfare workers, with the associated consequences for quality of life and ability to work, emphasizes the great importance of the long-term consequences of COVID-19 not only for those affected, but also for public health. The development of suitable therapy and rehabilitation concepts, especially for healthcare staff and other people at high risk, is therefore essential. Further research using standardized controlled designs is needed to investigate the pathomechanism, to further narrow down the core symptoms and to better distinguish between post-COVID symptoms and general effects of the pandemic.

## Notes

### Authors’ ORCID

- Albert Nienhaus: 0000-0003-1881-7302
- Matthias Bethge: 0000-0002-6417-7812
- Peter Koch: 0000-0003-2865-9483

### Ethics approval 

The study was conducted in accordance with the Declaration of Helsinki and approved by the Ethics Committee of the Hamburg Medical Association (protocol code 2021-10463-BO-ff, date of approval 16 March 2021). All participants gave their informed written consent to take part in the study.

### Funding

None.

### Availability of data and materials

The datasets used and analyzed during the current study are available from the corresponding author on reasonable request. 

### Acknowledgements

We would like to thank all participants and everyone involved from the BGW and UKE, without whom this study would not have been possible.

### Competing interests

The authors declare that they have no competing interests.

## Figures and Tables

**Table 1:**
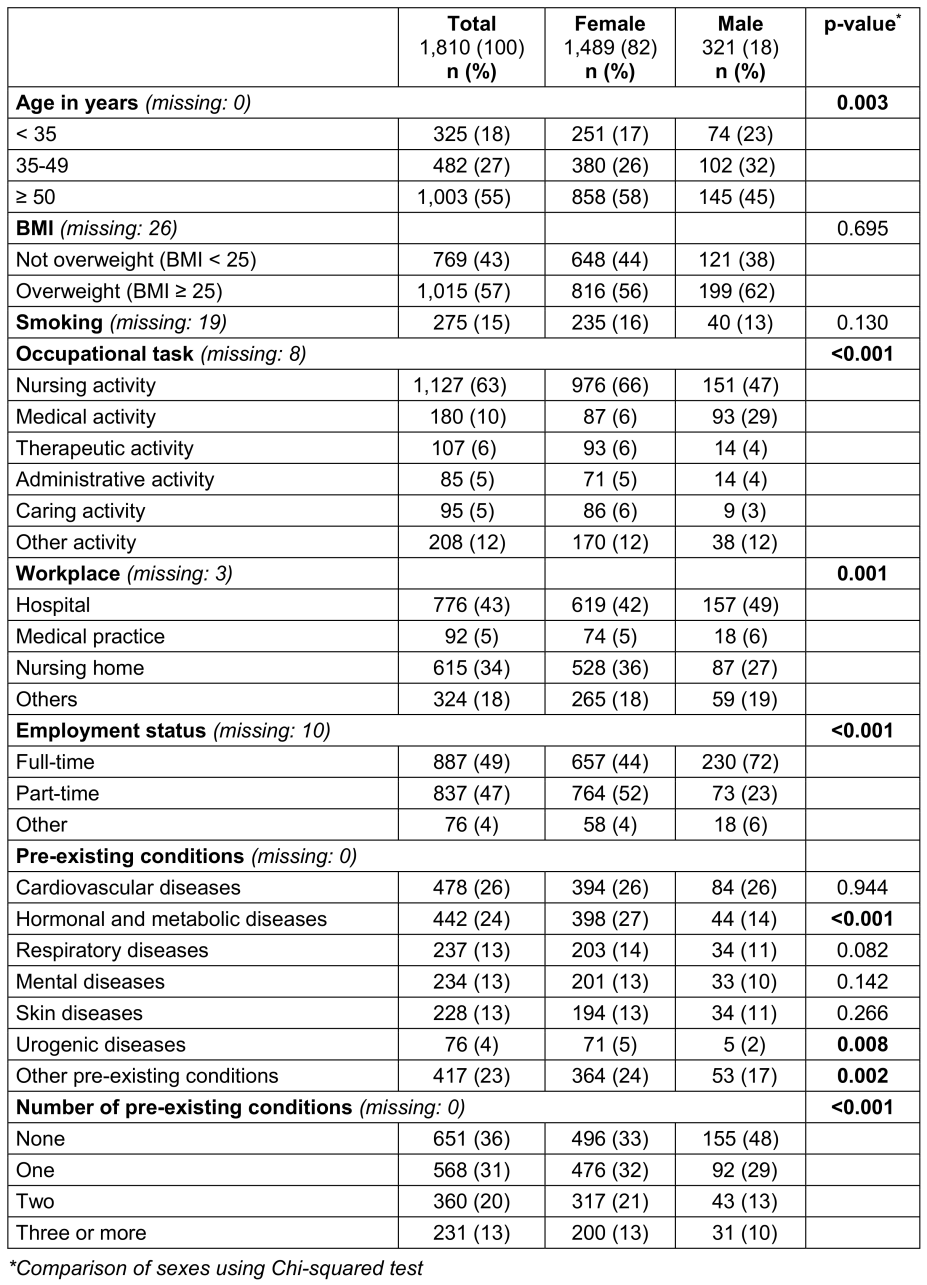
Participant characteristics by sex

**Table 2:**
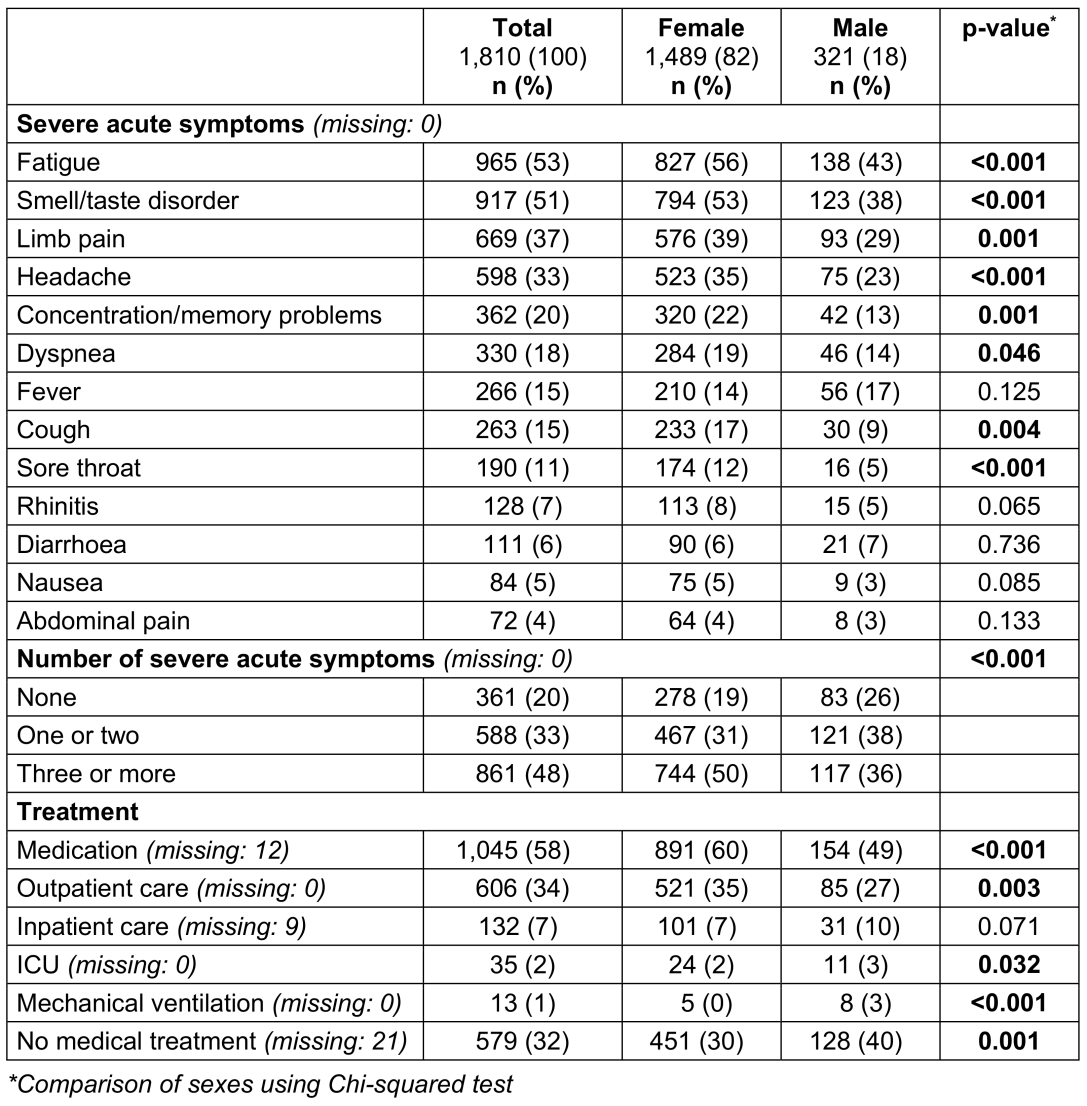
Characteristics of acute COVID-19 disease by sex

**Table 3:**
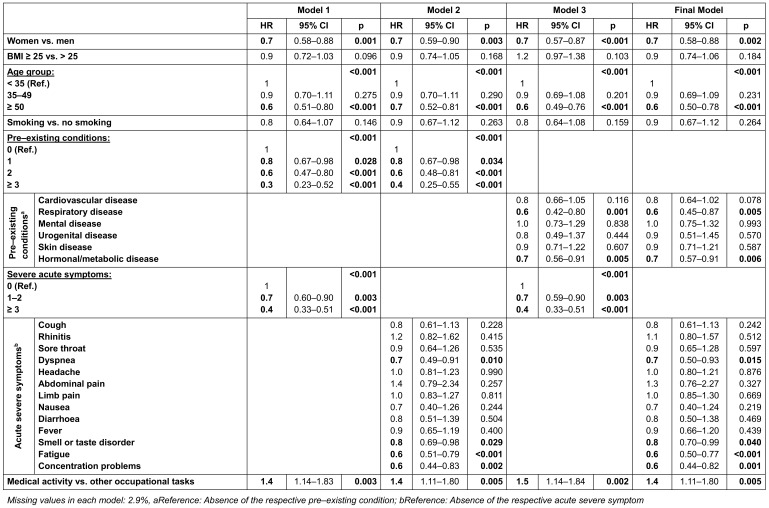
Models of the Cox regression
